# Adhesion and Invasion of Breast and Oesophageal Cancer Cells Are Impeded by Anti-LRP/LR-Specific Antibody IgG1-iS18

**DOI:** 10.1371/journal.pone.0066297

**Published:** 2013-06-18

**Authors:** Thandokuhle Khumalo, Uwe Reusch, Stefan Knackmuss, Melvyn Little, Robin B. Veale, Stefan F. T. Weiss

**Affiliations:** 1 School of Molecular and Cell Biology, University of the Witwatersrand, Johannesburg, Gauteng, The Republic of South Africa; 2 Affimed Therapeutics AG, Heidelberg, Baden-Wuerttemberg, Germany; The Scripps Research Institute Scripps Florida, United States of America

## Abstract

Adhesion and invasion have been identified as the two key components of metastasis. The 37 kDa/67 kDa laminin receptor (LRP/LR) is thought to enhance these two processes thus endorsing the progression of cancer. Here we report on LRP/LR and the metastatic potential of MDA-MB 231 breast and WHCO1 oesophageal cancer cells. Western blot analysis revealed a significant increase in total laminin receptor precursor (LRP) levels of breast and oesophageal cancer cells in comparison to non-invasive MCF-7 breast cancer cells, whereas LRP/LR cell surface levels in both cell lines were not significantly different to those of MCF-7 cells as analysed by flow cytometry. Incubation of breast and oesophageal cancer cells with the anti-LRP/LR specific antibody, IgG1-iS18, resulted in significant reduction in the adhesive potential of WHCO1 and MDA-MB 231 cells by 92% and 16%, respectively. Moreover, invasion was significantly impeded by 98% and 25% for WHCO1 and MDA-MB 231 cells, respectively. Pearson’s correlation coefficients proved a positive correlation between total LRP/LR levels and invasive potential as well as between the adhesive and invasive potential of breast and oesophageal cancer cells. Our findings suggest that through interference of the LRP/LR-laminin-1 interaction, anti-LRP/LR specific antibody IgG1-iS18 may act as a possible alternative therapeutic tool for metastatic breast and oesophageal cancer treatment.

## Introduction

Cancer has become a global burden due to its high incidence and mortality rates, with metastasis held accountable for approximately 90% of cancer deaths. According to the World Health Organization (WHO), cancer is the second leading cause of death amongst non-communicable diseases, claiming about 7.6 million lives in the year 2008. To date lung cancer is the most diagnosed cancer worldwide, followed by breast cancer, which is central to this study, in conjunction with oesophageal cancer noted as the eighth most diagnosed cancer (GLOBOCAN).

The 37-kDa/67-kDa laminin receptor (LRP/LR), a major receptor for extracellular matrix proteins, was first isolated from human breast carcinoma cells, murine melanoma cells [1] and normal muscle cells [2]. The relationship between the two isoforms, 37 kDa laminin receptor precursor and 67 kDa high affinity laminin receptor has not yet been encrypted but it is believed that the 37 kDa LRP isoform is the precursor of the 67-kDa LR possibly through acylation or heterodimerisation [3] rather than homodimerisation [4].

LRP/LR is found on the cell surface [5], the cytosol [6], [7]and nucleus [8], [9] and in the two latter cases it is involved in translational processes and maintenance of nuclear structures, respectively [3]. On the cell surface the receptor not only serves as a receptor for laminin but also acts as a co-receptor for elastin [10], carbohydrates [10] and the cellular prion protein [5], [11].

In its association with laminin-1, LRP/LR controls several physiological processes such as cell growth, adhesion, movement, differentiation and migration [12]. LRP/LR has also been implicated in numerous pathological processes such as facilitating the internalization of infectious prion proteins [13] and various viruses such as Dengue [14], Sindbis [14]and Adeno-associated viruses (AAVs) [15].

A direct association between the high levels of LRP/LR and the aggressiveness of tumorigenic cells was first noted in numerous cancer types, such as breast [16], cervical [17], [18], colon [18], [19], gastric [20], hepatocellular [21], lung [18], [22], ovarian [23], and prostate cancer cells [24]. However, knockdown of LRP using siRNAs resulted in decreased cell survival suggesting that LRP/LR is enhancing cell viability by blocking apoptosis [25]. Furthermore, recent findings demonstrated that anti-LRP/LR specific antibody W3 significantly impeded angiogenesis thus suggesting the LRP/LR might also be involved in tumor angiogenesis [26].

This correlation between high levels of LRP/LR and tumor aggressiveness indicates that the LRP/LR-laminin-1 interaction is pivotal for mediating the two key components of metastasis, adhesion and invasion [18], [27]. Cell adhesion allows the tumorigenic cell to adhere to the basement membrane that activates proteolytic enzymes i.e. type IV collagenase that degrade components of the extracellular matrix (ECM) such as laminins, proteoglycans and collagens [28]. Degradation of these components in turn induces invasion of the basement membrane, allowing the cancerous cell to migrate to a newly found microenvironment and proliferate there to form a secondary tumor [29].

The affiliation between LRP/LR levels and the aggressiveness of tumors recommends LRP/LR as a promising target for cancer treatment. This is supported by *in vivo* studies illustrating that high levels of LRP/LR result in tumor growth and proliferation [29]. Furthermore, we demonstrated that application of anti-LRP/LR specific antibodies scFv-iS18 and IgG1-iS18 on human fibrosarcoma (HT1080) cells results in decreased invasive potential of HT1080 cells [30]. Thus the hampering effect on invasion by anti-LRP/LR specific antibodies indicates interference of the LRP/LR-laminin-1 interaction.

In addition, we recently showed that anti-LRP/LR specific antibody IgG1-iS18 significantly reduced adhesion and invasion of the four most important cancer types worldwide, namely, cervical, lung, prostate, and colon cancer cells, suggesting that IgG1-iS18 might act as a powerful therapeutic tool for treatment of the above mentioned cancer types.

In this study, we investigated whether IgG1-iS18 is also capable of impeding adhesion and invasion of oesophageal and breast cancer cells, reflecting major cancer types worldwide. According to the most recent worldwide 2008 cancer statistics breast cancer was ranked the second most common cancer after lung cancer, with an estimated 1.38 million women diagnosed with the disease (Cancer Research UK). Furthermore, oesophageal cancer is graded as the eighth most common cancer, with an estimated 480 000 people diagnosed in 2008 (Cancer Research UK). These high incidence rates illustrate the crucial need for alternative therapeutic options for the treatment of these cancer types, both of them having only limited therapeutic options. Similar metastatic studies have been carried out in our laboratory, namely on fibrosarcoma, cervical cancer, lung cancer, prostate cancer and colon cancer cells, respectively, proving that anti-LRP/LR specific antibody IgG1-iS18 significantly reduces adhesion and invasion, major components of metastatic cancer [18], [30]. However, to prove that IgG1-iS18 is also effective on breast and oesophageal cancer cells, major emerging cancer types worldwide, we conducted this study to offer a possible alternative therapeutic antibody option for patients suffer from these cancer types.

## Materials and Methods

### Cell Culture and Conditions

Human breast adenocarcinoma (MCF-7) cells were cultured in Dulbecco’s modified Eagle’s medium (DMEM) high glucose (4.5 g/l) (Invitrogen Gibco). MDA-MB 231 breast cancer cells were cultured in DMEM/Ham’s-F12 (1∶1) and WHCO1 oesophageal cancer cells were cultured in DMEM/Ham’s-F12 (3∶1). All media was supplemented with 10% fetal calf serum (FCS) and 1% penicillin/streptomycin at 37°C and 5% CO_2_.

The oesophageal WHCO1 carcinoma cell line is not commercially available. This epithelial cell line was propagated *in vitro* from tumor biopsy material of a patient with this type of oesophagus carcinoma [31]. Cell lines MCF-7 and MDA-MB-231 are commercially available at the American Tissue Culture Collection (ATTC), catalogue numbers ATCC® HTB22™ and ATCC ® HTB-26™, respectively.

### Reagents and Antibodies

Matrigel™, employed for cell invasion assays is derived from the Engelbreth-Holm-Swarm (EHS) mouse sarcoma and was obtained from BD Biosciences.

Laminin-1, employed for adhesion assays was obtained from Sigma-Aldrich.

Chloramphenicol acetyl transferase (CAT) antibody was obtained from Sigma-Aldrich.

IgG1-iS18 was recombinantly produced in a mammalian expression system as described by Zuber et al., (2008) [30].

### SDS PAGE and Western Blotting

Western blotting was employed in order to determine total LRP/LR levels in the various cancer cell lines. 10 µg of total protein was subjected to sodium dodecyl sulfate polyacrylamide gel electrophoresis (SDS-PAGE) and the resulting protein pattern was transferred onto a polyvinylidine fluoride (PVDF) membrane with 1X transfer buffer at 450 mV for 45 min. Post blocking with non-fat milk and PBS Tween, the membrane was probed with IgG1-iS18 primary antibody (1∶10000) for an hour then washed three times with PBS Tween prior to incubation with goat-anti-human-peroxidase (1∶5000) secondary antibody. In addition, 42-kDa β-actin was used as a loading control by using the monoclonal anti-β-actin-peroxidase antibody (1∶5000). The resulting fluorescence of the horseradish peroxidase (HRP) was developed and fixed onto an X-ray film. Data shown is representative of three biological replicates.

### Flow Cytometry - FACS™ Analysis

Cell surface expression of LRP/LR was determined using a flow cytometer. Two cell suspensions were prepared by re-suspension in FACS buffer composed of 0.01% sodium azide, 2% fetal calf serum, 20 mM ethylenediaminetetraacetic acid (EDTA) and phosphate-buffered saline (PBS). One cell suspension was stained for LRP/LR by re-suspension in 100 µl of 30 µg/ml of primary antibody IgG1-iS18, whilst the second cell suspension posing as the control re-suspended in fluorescence activated cell scanning (FACS) buffer only. Following 1 hour (h) incubation in the dark, cell pellets were washed three times and re-suspended in 100 µl of 30 µg/ml the goat anti-human IgG fluorescein isothiocyanate (FITC)-coupled secondary antibody (Beckman Coulter). After 1 h incubation, suspensions were centrifuged at 5000 rpm for 1 minute (min) and pellets washed three times with FACS buffer. The resulting suspensions were then evaluated using the BD Accuri C6 flow cytometer and software. Data shown is representative of three biological replicates.

### Invasion Assay

The ability of the cell lines to invade the basement membrane was examined *in vitro*, using the ECM-like Matrigel™. The Matrigel™ was diluted in serum free-cold cell culture media, loaded on to inserts of 24-transwell plates (BD Falcon, 8 µm pore size) and allowed to solidify at 37°C for approximately 5 hours. Harvested cells were re-suspended in serum-free culture media at density of 1×10^6^ cells/ml, loaded onto the Matrigel™ and incubated for an 18 h period at 37°C prior to assessment. Furthermore, to examine the influence of the anti-LRP/LR antibody, cells were incubated with the antibodies, IgG1-iS18 (0.2 µg/µl) and anti-CAT (0.2 µg/µl, negative control), with 500 µl of culture media added to the lower chamber. After 18 h incubation, non-invasive cells were removed by a cotton swab and invasive cells were washed with PBS, fixed with 4% paraformaldehyde (PFA) and stained with 0.5% toulidine blue. Absorbance was then measured with an enzyme-linked immunosorbent assay (ELISA) reader at 620 nm following extraction of the dye with 1% SDS. Data shown is representative of three biological replicates.

### Adhesion Assay

Analysis of the adhesive potential of the various cancer cell lines to the basement membrane was performed *in vitro* using 96-well plates coated with laminin (10 µg/ml), and uncoated wells posing as negative controls. Following coating of wells for 1 h, wells were washed with 0.1% bovine albumin serum (BSA) in DMEM and blocked with 100 µl of 0.5% BSA in DMEM for an hour. Cells suspended in serum-free culture media were added to wells at a density of 4×10^5^ cells/ml in order to examine the adhesive potential. Furthermore, the effect of the anti-LRP/LR IgG1-iS18 (0.2 µg/µl) antibody on the adhesive potential was analysed (CAT antibody acting as a negative control). Cell suspensions treated with either antibody were incubated for one hour, washed and fixed with 4% PFA for 10 min. Wells were then stained with 0.1% crystal violet, followed by the addition of 1% SDS in order to extract the dye and the absorbance of the resulting colorimetric solution was measured at 570 nm. Data shown is representative of three biological replicates.

### Statistical Evaluation

Data was statistically analyzed using the two-tailed Student’s *t-test* with a confidence interval of 95%; with p-values less than 0.05 considered significant.

The Pearson’s (r) correlation coefficient was used to measure the degree of association between LRP/LR levels and the adhesive/invasive potential. A positive coefficient indicated direct proportionality between the two variables, thus a negative value suggested inverse proportionality.

## Results

### Breast and Oesophageal Cancer Cells Display High Levels of LRP/LR on the Cell Surface

The interaction of LRP/LR and laminin-1 on the cell surface of tumorigenic cells triggers the process of metastasis. Therefore, we assessed the percentage of breast and oesophageal cancer cells exhibiting LRP/LR on the cell surface.

**Figure 1:**
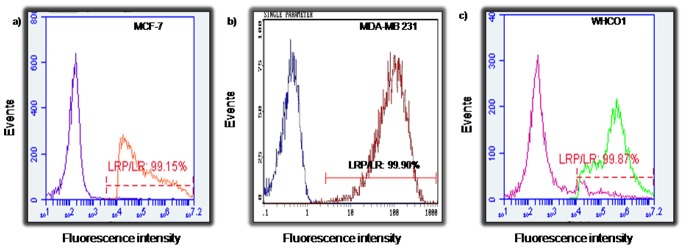
Detection of LRP/LR levels on the surface of oesophageal and breast cancer cells. FACS™ was performed using IgG1-iS18 antibody on (a) MCF-7, (b) MDA-MB 231 and (c) WHCO1 cells. The first curve represents the control labeled with goat anti-human IgG-coupled secondary antibody whilst the second curve represents cells labeled with both anti-LRP IgG1-iS18 primary and goat anti-human IgG-coupled secondary antibodies (30 µg/ml). Percentages were calculated using a linked marker from the point of overlap to the end of the second curve. Data are representative of three experiments carried out in triplicate using different cell cultures.

Both tumorigenic cell lines revealed high levels of LRP/LR on the cell surface as shown in Figure 1. 99.90% of MDA-MB 231 breast cancer cells (Fig. 1b) and 99.87% of WHCO1 oesophageal cancer cells revealed LRP/LR on the cell surface (Fig. 1c). We confirmed previous studies that non-invasive MCF-7 cells reveal high levels of LRP/LR [18].

### Breast and Oesophageal Cancer Cells Display High Total LRP/LR Levels

**Figure 2:**
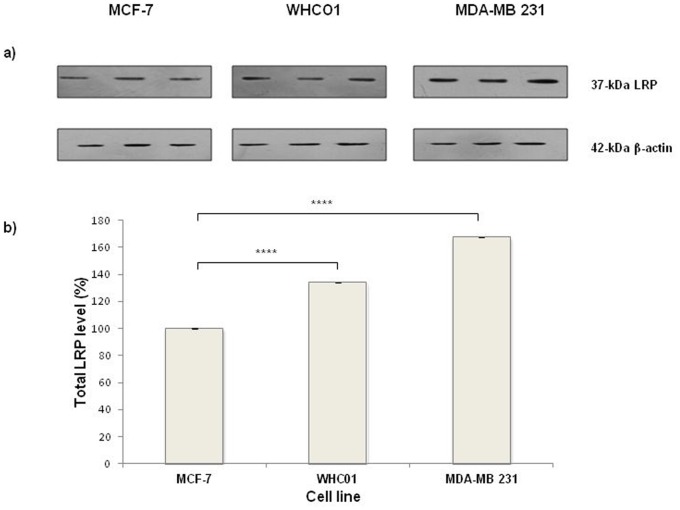
Detection of total LRP levels in oesophageal and breast cancer cells. (a) Detection of relative expression of the total 37 kDa LRP levels on protein lysates of various tumorigenic cell lines. Western blot analysis was performed using anti-LRP/LR specific primary antibody IgG1-iS18 and secondary FITC-coupled antibody on MCF-7, WHCO1 and MDA-MB 231 cells. β-actin was used as a loading control and 10 µg of protein lysates was loaded. Data are representative of three experiments carried out in triplicate using different cell cultures. (b) Bar chart of total LRP/LR levels of MCF-7, WHCO1 and MDA-MB 231 cell as analysed by Western blotting. Densitometry analysis was performed on the blots from Fig. 2a. WHCO1 and MDA-MB 231 cells displayed significantly higher total LRP/LR levels relative to MCF-7 cells. Data are representative of three experiments carried out in triplicate from different cell cultures.

Since LRP/LR is also found intracellularly [9] we determined total LRP/LR levels. All tumorigenic cell lines revealed expression of the 37 kDa laminin receptor precursor (LRP) (Figure 2a). The 67 kDa high affinity laminin receptor (LR) could not be detected with IgG1-iS18 as previously described [30].

Densitometry analysis revealed that MDA-MB 231 and WHCO1 tumorigenic cell lines have significantly higher total LRP levels relative to non-invasive MCF-7 cell line (Figure 2b). MDA-MB 231 and WHCO1 cells revealed a significant 67.3% and 33.8% increase in total LRP levels, respectively, compared to MCF-7 cells (Fig. 2b).

### The Invasive Potential of Oesophageal and Breast Cancer Cells is Significantly Higher than Non-invasive MCF-7 Cells

**Figure 3:**
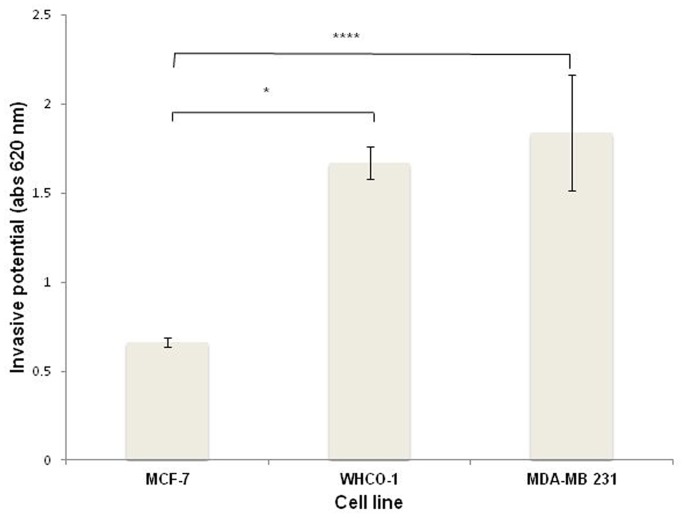
Oesophageal and breast cancer cells display significantly increased invasive potential compared to non-invasive MCF-7 cells. Cells were incubated in Matrigel™ coated inserts for an18 h period and stained with toulidine. The dye was extracted using SDS and measured the absorbance of the resulting solution at 620 nm. Data are representative of three experiments carried out in triplicate.

Invasion is regarded as a pre-requisite for the progression of cancer thus the ability of breast and oesophageal cancer cells was analysed. The known non-invasive MCF-7 cell line revealed very little invasive potential (Fig. 3). Whilst the WHCO1 and MDA-MB 231 cells revealed a significant 60% and 71% increase in invasive potential, respectively when compared to MCF-7 cells (Table 1).

**Table 1:** Invasive potential of oesophageal and breast cancer cells.

Cell line	Invasive potential	% Increase of Invasive potential compared to MCF7-cells
**MCF-7**	Non-invasive	–
**WCHO1**	Invasive	60% (p = 0.016)*
**MDA-MB 231**	Invasive	71% (p = 0.0002)**

Invasive potentials were determined using the absorbance measured at 620 nm. A percentage was then determined by calculating the difference between the respective cell line and the invasive potential of MCF-7 cell line. p-values were calculated using the two-tailed Students t-test with a 95%confidence interval.

### IgG1-iS18 Significantly Impedes the Adhesion Potential of Breast and Oesophageal Cancer Cells

**Figure 4:**
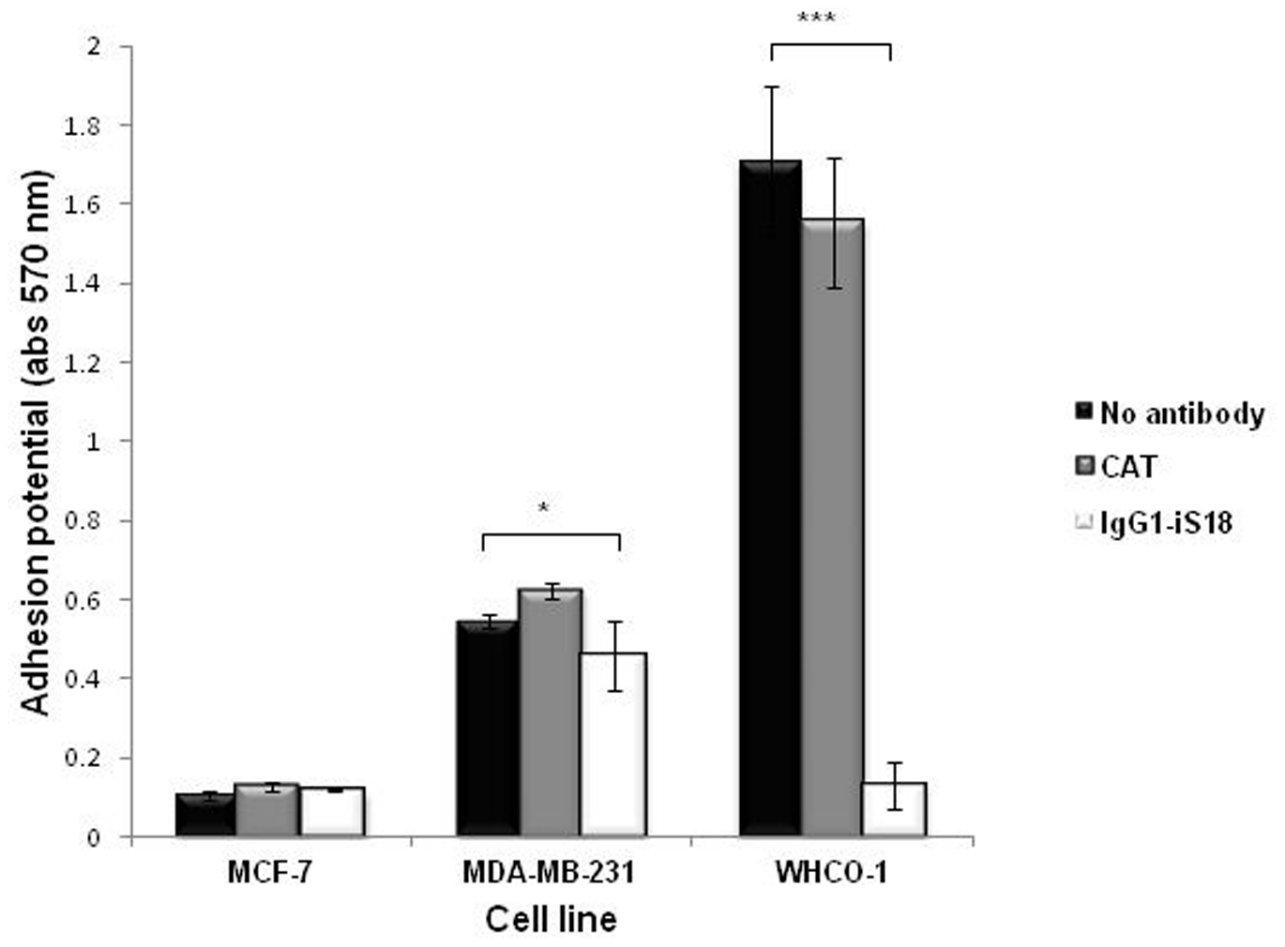
Effect of anti-LRP/LR specific antibody IgG1-iS18 on the adhesive potential of breast and oesophageal cancer cells. Cells were incubated with IgG1-iS18 (0.2 mg/ml) and CAT acting as a negative control (0.2 mg/ml) for an hour on laminin-1 coated wells. Cells that had adhered were stained with crystal violet and absorbance of extracted dye was measured at 570 nm. A significant decrease in adhesion potential was noted for both WHCO1 and MDA-MB 231 cells upon application of IgG1-iS18 (light gray) compared to the no antibody control (black). Data are representative of three experiments carried out in triplicate from different cell cultures.

Adhesion of a tumorigenic cell to the basement membrane via the laminin-1-LRP/LR interaction is crucial for initiating invasion as it facilitates other interactions that are pivotal for degradation of the components of the basement membrane. Thus the adhesive potential of breast and oesophageal cancer cells was analysed and the effect of anti-LRP/LR specific antibody IgG1-iS18 on the adhesive potential was assessed. Upon application of IgG1-iS18 antibody, a significant decrease in the adhesive potential was noted in both neoplastic cell lines, but no effect was observed on the minimal adhesion potential of non-invasive MCF-7 cells (Fig. 4). The oesophageal WHCO1 cancer cells displayed the highest adhesive potential, accompanied by the highest decrease of adhesive potential of 92% whilst only a 16% decrease was observed for the MDA-MB 231 breast cancer cells (Table 2). Furthermore, the control antibody CAT had no effect on the adhesive potential of all tumorigenic cell lines.

**Table 2:** IgG1-iS18 reduces adhesion potential of breast and oesophageal cancer cells.

Cell line	% Reduction in adhesion potential	p-value
**MCF-7**	0	–
**WHCO1**	92	0.0002**
**MDA-MB 231**	16	0.0124*

The % reduction in adhesion is the difference in adhesive potential between cells without antibody compared to cells treated with IgG1-iS18 antibody. p-values were calculated using the two –tailed Students *t-test* with a 95% confidence interval.

### IgG1-iS18 Significantly Hampers Invasion of Breast and Oesophageal Cancer Cells

**Figure 5:**
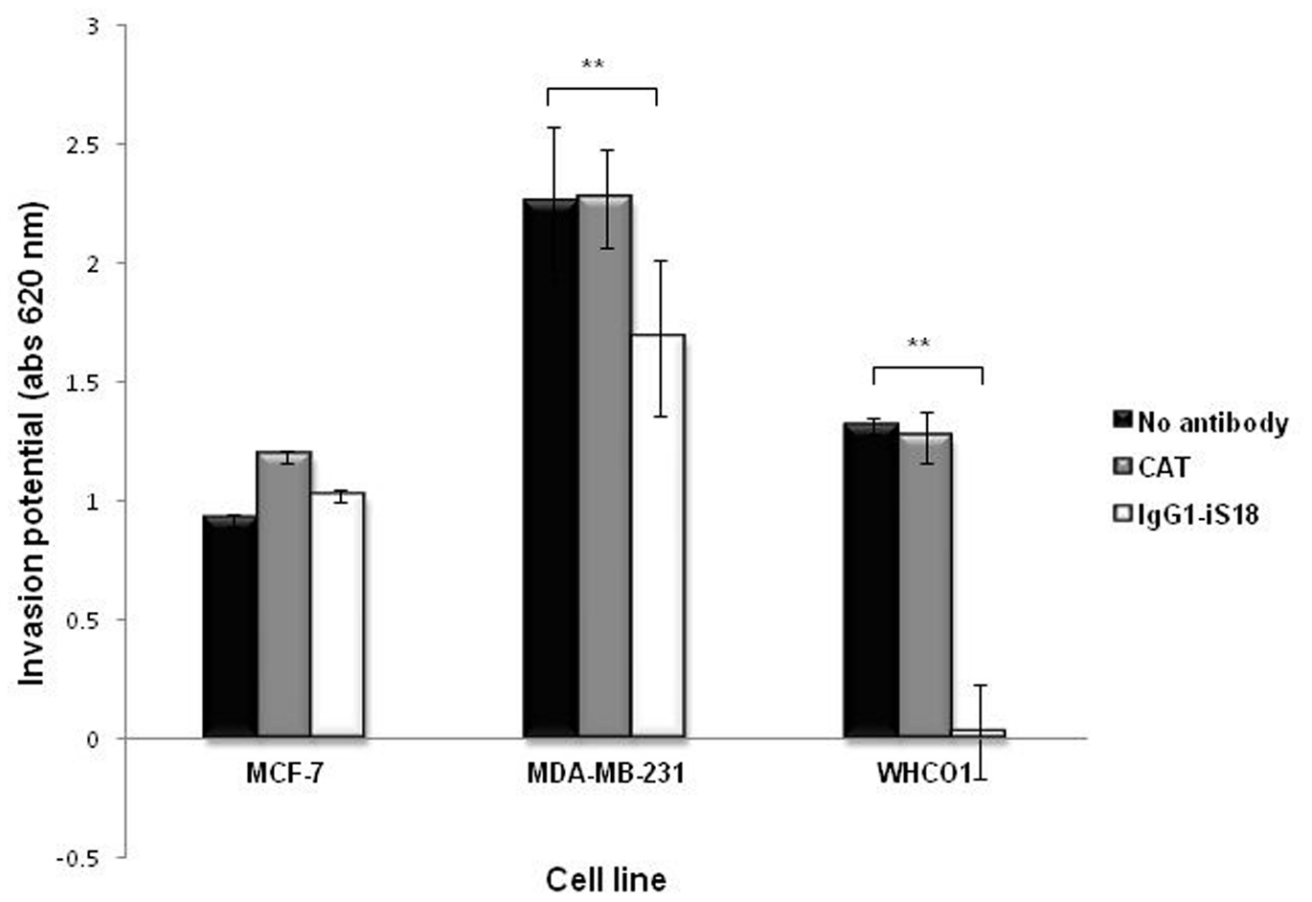
Effect of anti-LRP/LR specific antibody IgG1-iS18 on the invasive potential of breast and oesophageal cancer cells. Cells were incubated with IgG1-iS18 (0.2 mg/ml) or CAT (negative control) (0.2 mg/ml) on Matrigel™ coated inserts for an 18 h period. Toulidine blue was used to stain the invaded cells and following extraction of the dye using SDS absorbance was measured at 620 nm. A significant decrease in invasion potential was noted for both WHCO1 and MDA-MB 231 cells upon application of IgG1-iS18 antibody (light gray) compared to the no antibody control (black). Data are representative of three experiments carried out in triplicate from different cell cultures.

In order to ascertain the effect of the anti-LRP/LR specific antibody on the invasion of the tumorigenic cell lines, cells were incubated with IgG-iS18. A significant decrease of the invasion was observed for the WHCO1 and MDA-MB 231 cell lines (Fig. 5). A more striking decrease was noted for the WHCO1 (human oesophagus carcinoma) cell line (Fig. 5 and Table 3), concurrent with the highest decrease in adhesive potential observed in this cell line (Fig. 4). However, only a 25% decrease was observed for the MDA-MB 231 (breast cancer carcinoma) cell line which is also noted as the most aggressive cell line with a 71% increase in invasion potential (Table 1).

**Table 3:** IgG1-iS18 reduces invasive potential of breast and oesophageal cancer cells.

Cell line	% Reduction in invasion potential	p-value
**MCF-7**	0	–
**WHCO1**	98	0.0084**
**MDA-MB 231**	25	0.0083**

The % reduction in invasion in the difference in invasive potential between cells without antibody compared to cells treated with IgG1-iS18 antibody. p-values were calculated using the two-tailed Students *t-test* with a 95% confidence interval.

## Discussion

Numerous studies have pointed out that the LRP/LR-laminin-1 interaction may be crucial for malignant transformation of tumorigenic cells and angiogenesis induction [32]. Omar *et al*. [18] have shown that the anti-LRP/LR specific antibody IgG1-iS18 significantly hampers the two key steps of metastasis, adhesion and invasion. Here the effect of this antibody on the adhesive and invasive potential of MDA-MB 231 breast and WHCO1 oesophageal cancer cell lines was investigated.

A remarkably high percentage of all tumorigenic cell lines namely, MCF-7, WHCO1 and MDA-MB 231 (99.15%, 99.87% and 99.99%, respectively) displayed LRP/LR on their cell surface. As formerly stated, LRP/LR is pivotal for the cell adhesion, invasion, migration and proliferation. Both MDA-MB 231 and WHCO1 tumorigenic cells are known to be invasive [33], [34], thus the high proportion of cells displaying LRP/LR on the cell surface may be owing to the high invasive potential of these cells. Although approximately the same percentage of WHCO1 and MDA-MB 231 cells show LRP/LR on the cell surface, WHCO1 cells show less invasive potential compared to MDA-MB 231 cells (Fig. 3).

In addition to confirmation of the cell surface expression of this receptor, Western blot analysis (Fig. 2) was also employed to analyse the total LRP levels and indeed all three tumorigenic cell lines expressed the laminin receptor. By means of densitometry analysis, significantly increased total LRP levels on oesophageal (WHCO1) and breast (MDA-MB 231) cancer cells compared to non-invasive MCF-7 cells were observed (Fig. 2a).

Apart from conferring the main metastatic characteristics (adhesion, invasion and migration), LRP/LR is also implicated in protein synthesis and angiogenesis induction [32], [35]. Thus the high levels of total LRP noted in WHCO1 and MDA-MB 231 cell lines may be owing to the metastatic origin of these cell lines. These cell lines may require enhanced protein synthesis in order to carry out the metastatic processes central to cancer progression and especially induce the formation of new blood vessels (angiogenesis) thus sustaining growth of the cancer due to availability of ample nutrients [28]. Therefore the elevated levels of LRP on these cell lines are not puzzling in cancer terms, and may assist in pathways that are central in metastasis.

Furthermore, the breast cancer MDA-MB 231 cell line is a triple negative form of breast cancer since these cells do not express estrogen (ER), progesterone (PR) and Human Epidermal Growth Factor Receptor 2 (HER2) [36], [37]. Thus it has been shown that progression of this cancer cell line is independent of the presence of these hormones, therefore the high levels of LRP/LR observed in the MDA-MB 231 cell line may be an alternative mechanism for survival, growth and progression. The upregulation of receptors is characteristic of a majority of cancer types, and triple negative cancers are also of no exception as studies have shown an overexpression of the Epidermal Growth Factor Receptor (EGFR) and numerous cytokeratins amongst these cancer cells [38] thus this overexpression of LRP/LR is not eccentric.

It is noteworthy to add that the correlation between the levels of total LRP/LR and the invasive potential of both cell lines was considerably high (Table 4), signifying a positive, directly proportional relationship between the two parameters. Therefore, suggesting that cancer aggressiveness is enhanced by high LRP/LR levels, which is consistent with results obtained by Omar *et al*. [18]. Please note that only the total LRP/LR levels were compared to the invasive potential because no significant difference in cell surface levels was observed between tumorigenic and non-tumorigenic cells (Fig. 1).

**Table 4:** Pearson’s correlation co-efficient.

Cell line	Total LRP/LR levels to invasive potential	Adhesive potential to invasive potential
**MDA-MB 231**	0.95	0.97
**WHCO1**	0.98	0.96

The results obtained in this study demonstrate that anti-LRP/LR specific antibody, IgG1-iS18, not only considerably decreased the adhesion of metastatic oesophageal and breast cancer on laminin-1 but also reduced the invasive capability of these cells on the Matrigel™. The extent of inhibition of these processes *in vitro* varied for each cell line (Table 2 &3) with a striking decrease observed for the oesophageal WHCO1 cell line and these discrepancies may be attributed to varying total LRP/LR levels (Fig. 3). This significant decrease in invasive potential upon IgG1-iS18 application may be accredited to the inhibition of adhesion, as this step is preceding invasion during metastasis induction.

We suggest from the high correlation coefficient between invasive potential and adhesion potential on breast and oesophageal cancer cells of 0.97 and 0.96, respectively (Table 4), that invasion is correlated with the laminin-1/LRP/LR interaction and therefore we suggest that the significant reduction in the invasive potential by IgG1-iS18 is due to a blockage of the LRP/LR laminin-1 interaction. However, we cannot exclude that the significant reduction in invasion of both cell lines by IgG1-iS18 might also be due to a reduced level of LRP/LR on the cell surface.

This result is in accordance with reported results, affirming that the LRP/LR-laminin-1 interaction not only facilitates adhesion but promotes the secretion of basement degrading enzymes such as type IV collagenase, thus promoting invasion and migration through the newly formed channels [33].

In addition, the pathological potential of the LRP/LR-laminin interaction has been targeted using other anti-LRP/LR tools such as heparan mimetic HM2602, pentosan polysulfates and antibodies, single-chain variable fragment (scFv) and IgG1-iS18, resulting in a significant obstruction of the adhesive and invasive potential of HT1080 human fibrosarcoma cells [30]. Furthermore, another study observed hindrance of both adhesive and invasive potential of AMC-HN-8 laryngeal carcinoma cells upon application of a specific anti-67LR monoclonal antibody, MluC5 [39]. Thus the hampering effect on adhesion and invasion by these anti-LRP/LR tools indicates interference with the laminin-1-LR/LRP interaction.

Taken together, these results strongly suggest that the LRP/LR-laminin-1 interaction in tumor cells enhances the proteolytic cleavage of the basement membrane, thus facilitating invasion and migration [40]. Additionally, the anti-LRP/LR specific antibody IgG1-iS18 also significantly impacted on the behavior of WHCO1 and MDA-MB 231 cancer cells at the stages of adhesion and invasion of the basement membrane and may potentially act as an alternative therapeutic tool for the treatment of metastatic oesophageal and breast cancer.

The results of this study will provide new insight in the scientific community because one cannot speculate that the IgG1-iS18 antibody will have the same hindering influence on all cancer types as each cancer behaves differently. Moreover, targeting LRP/LR may prove difficult in terms of administration because as previously mentioned, it is also imperative for numerous physiological processes thus its inhibition may cause further deleterious effects that can compromise the health of the individual. Therefore studies into potentially appropriate delivery systems could assist in this quandary and subsequent to successful animal trials this antibody could indeed be a potential therapeutic tool and contribute in the quest to fight cancer.
